# Isoproterenol-Induced Cardiomyopathy Recovery Intervention: Amlexanox and Forskolin Enhances the Resolution of Catecholamine Stress-Induced Maladaptive Myocardial Remodeling

**DOI:** 10.3389/fcvm.2021.719805

**Published:** 2021-11-25

**Authors:** Gabriel Komla Adzika, Hongjian Hou, Adebayo Oluwafemi Adekunle, Ruqayya Rizvi, Joseph Adu-Amankwaah, Wenkang Shang, Kexue Li, Qi-Ming Deng, Richard Mprah, Marie Louise Ndzie Noah, Hong Sun

**Affiliations:** ^1^Department of Physiology, Xuzhou Medical University, Xuzhou, China; ^2^The College of Biology and Food, Shangqiu Normal University, Shangqiu, China; ^3^Department of Clinical Medicine, Xuzhou Medical University, Xuzhou, China; ^4^Institute for Biochemistry and Molecular Biology, ZBMZ, Faculty of Medicine, Albert-Ludwigs University Freiburg, Freiburg, Germany; ^5^Faculty of Biology, Albert-Ludwigs University Freiburg, Freiburg, Germany; ^6^The Key Laboratory of Cardiovascular Remodeling and Function Research, The State and Shandong Province Joint Key Laboratory of Translational Cardiovascular Medicine, Department of Cardiology, Chinese Ministry of Education, Chinese National Health Commission and Chinese Academy of Medical Sciences, Qilu Hospital of Shandong University, Jinan, China

**Keywords:** stress, amlexanox, forskolin, isoproterenol-induced cardiomyopathy, left ventricular systolic dysfunction, myocardial inflammation, myocardial fibrosis

## Abstract

The increasing incidence of stress-induced cardiomyopathy is due to the complexities of our modern-day lives, which constantly elicit stress responses. Herein, we aimed to explore the therapeutic potential of Amlexanox and Forskolin in promoting the recovery from stress-induced cardiomyopathy. Isoproterenol-induced cardiomyopathy (ICM) models were made, and the following treatment interventions were administered: 5% v/v DMSO as a placebo, Amlexanox (2.5 mg/100 g/day) treatment, Forskolin (0.5 mg/100 g/day), and Amlexanox and Forskolin combination, at their respective aforementioned dosages. The effects of Amlexanox and Forskolin treatment on ICM models were assessed by eletrocardiography and echocardiography. Also, using histological analysis and ELISA, their impact on myocardial architecture and inflammation were ascertained. ICM mice had excessive myocardial fibrosis, hypertrophy, and aggravated LVSDs which were accompanied by massive CD86+ inflammatory cells infiltration. Amlexanox treatment attenuated the myocardial hypertrophy, fibrosis, and inflammation and also slightly improved systolic functions. Meanwhile, forskolin treatment resulted in arrhythmias but significantly enhanced the resolution of myocardial fibrosis and inflammation. Intriguingly, Amlexanox and Forskolin combination demonstrated the most potency at promoting the recovery of the ICM from LVSD by attenuating maladaptive myocardial hypertrophy, fibrosis, and inflammatory responses. Our findings highlight the Amlexanox and Forskolin combination as a potential therapeutic intervention for enhancing cardiac function recovery from stress-induced cardiomyopathy by promoting the resolution of maladaptive cardiac remodeling.

## Introduction

The incidence of cardiovascular diseases (CVDs) keeps increasing due to the enormous amount of risk factors (sex-gender difference, heredity, and unhealthy lifestyles) which hastens their progression. Typically, stress resulting from the demands and complexities of our modern-day lives adversely affects overall cardiac health if it remains chronic (1–4). During chronic stress, elevated levels of circulating catecholamine overstimulate β-adrenergic receptors (βARs), which induces their dysregulation and causes the initiation of cardiomyopathies (1, 5, 6). Recent studies have associated the development of hypertrophied hearts and left ventricular systolic dysfunctions (LVSD) with chronic stress-induced cardiomyopathy (7).

Isoproterenol (an agonist of β_1_AR and β_2_AR), besides being used for bradycardia treatment, has been extensively used to mimic catecholamines in modeling chronic stress-induced cardiomyopathy [hereafter referred to as Isoproterenol-induced cardiomyopathy (ICM)] so as to be able to elucidate its underlying pathomechanisms (8, 9).

Despite considerable efforts in elucidating the disease mechanisms of ICM, which have mainly implicated the maladaptive stimuli signal mediation of β_2_AR (5), very few treatment interventions aimed at attenuation of the pathological cardiac remodeling have been demonstrated in animal models. Conversely, there are overwhelming numbers of studies demonstrating potential therapeutic intervention for acute myocardial infarction (AMI). Like AMI, ICM is characterized by exacerbated myocardial inflammation and LVSD (7, 10, 11). Hence, this study sought to explore if amlexanox (AMLX), which was recently demonstrated to aid in the recovery of cardiac function from AMI, could do the same in ICM models. Also, cyclic adenosine monophosphate (cAMP) plays essential roles in modulating cardiac and inflammatory responses adaptively. However, cAMP is reported to be downregulated in most CVDs (1, 12); hence forskolin (FSKN) was utilized as a treatment intervention to ensure its bioavailability and explore the possible therapeutic outcomes.

Herein, we demonstrated that AMLX and FSKN combination is the most potent at attenuating the progression of LVSD and enhancing cardiac function recovery in ICM models. We also showed that this treatment intervention facilitates cardiac fibrosis resolution and adaptively modulates myocardial inflammatory.

## Materials and Methods

### Experimental Animals and Models

Mice (FVB males aged 2–3 months) were randomly grouped for *in vivo* experiments. 0.5 mg/100 g/day of ISO were injected subcutaneously (s.c.) as done previously (2, 13), for 21 days to induce ICM in the mice. The 5% v/v dimethyl sulfoxide equivalents were injected (s.c.) to the placebo (Pb) group.

**Figure 1:**
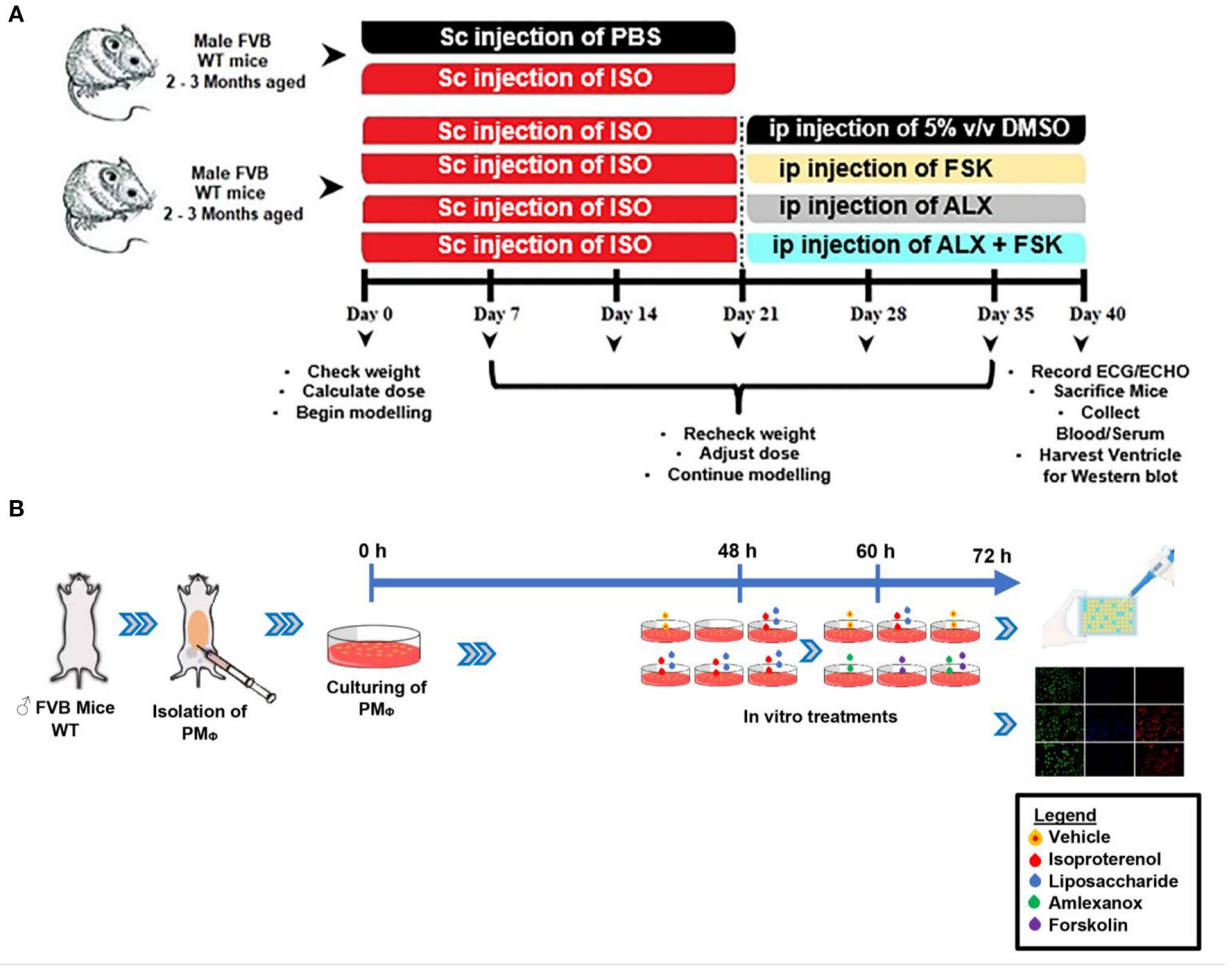
**(A,B)** Schematic of the experiment design timeline for making *in vivo* and *in vitro* models, respectively. ISO, Isoproterenol; Sc, Subcutenous; ip, intraperitoneal; FSK, forskolin; ALX, Amlexanox.

ICM recovery was initiated after day 21 of ISO administration. The recovery groups included; placebo treatment (PbT) [5% v/v dimethyl sulfoxide (DMSO)], 2.5 mg/100 g/day of AMLX (Abcam; ab142825) treatment and 0.5 mg/100 g/day of FSKN (Tocris Bioscience, UK; 1099) treatment. Also, AMLX and FSKN combine treatments were employed (Figure 1A). The dosages of AMLX and FSKN employed were based on their efficacies demonstrated in previous studies (2, 11).

Euthanization of mice for heart and peritoneal macrophage (PM_Φ_) isolations were done by cervical dislocation.

### Electrocardiography and Echocardiography

Mice (*n* = 6–8 per treatment group) were sedated with 0.5% isoflurane and secured with echo gel. The body temperatures were maintained at 37°C to enable electrocardiography (ECG) data acquisition using Vevo 2100 Ultrasound system (VisualSonics, Canada). Simultaneously in M-mode, systolic cardiac function indexes; Ejection fraction (EF), and fractional shorten (FS) were assessed as previously described (14). Also, left ventricle internal diameters, posterior wall, and interventricular septal wall thicknesses were assessed at end-systole and end-diastole.

### Histological Analysis of Hypertrophy, Interstitial Fibrosis, and Immune Cells Infiltration

Excised hearts (*n* = 6–8 mice per group) were rinsed, fixed overnight in 10% formaldehyde, embedded in paraffin, and sectioned. Wheat germ agglutinin (WGA) (Thermo Fisher; W11261), Masson's trichrome (Solarbio; G1340), and H&E (Solarbio; G1120) staining were done to ascertain cardiomyocyte hypertrophy and interstitial fibrosis deposition. Also, CD68 (Abcam; ab955), CD86 (Abcam; ab53004), and CD206 (Abcam; ab8918) immunohistochemical (IHC) staining were then performed to evaluate myocardial inflammatory cells infiltration. Microscopies were performed at x400 magnification and analyzed using ImageJ (1.53 h version; National Institute of Health).

### Enzyme-Linked Immunosorbent Assay

Sera (*n* = 4–8 mice per group) were used to evaluate concentrations of inflammatory mediators and cytokines; iNOS (JL20675; Jianglai Bio, Shanghai), Arg-1 (JL13668; Jianglai Bio, Shanghai), IL-1β (Abcam; ab197742), IL-6 (Abcam; ab222503), TNFα (Abcam; ab208348), IL-10 (Abcam; ab255729), TGF-β (Proteintech; KE10005). cAMP bioavailability was assessed with cAMP ELISA kits (JL13362; Jianglai Bio, Shanghai). Also, myocardial lysates (*n* = 6–8 mice per group) were used to evaluate ANP (JL20612; Jianglai Bio, Shanghai) and BNP (JL12884; Jianglai Bio, Shanghai) concentrations. The assays were performed as per the manufacturer's instructions and in triplicates.

### Western Blot

Concentrations of lysates obtained from apical myocardia (*n* = 4 hearts per group) were normalized, treated with loading buffer, and proteins denatured at 100°C for 10 min. The proteins were separated were SDS-PAGE gels, transferred on PVDF membranes which were blocked and blotted overnight with the following antibodies: Collagen Type I (Proteintech; 14695-1-AP), Collagen Type III (Proteintech; 13548-1-AP), ANP (Santa Cruz Biotechnology; sc-515701), BNP (Santa Cruz Biotechnology; sc-271185), Cleaved Caspase-3 (Cell Signaling Technology; 9661T) and GAPDH (Proteintech; 10494-1-AP). Western blots were performed in triplicates and normalized with their respective loading controls.

### Immune Cells Isolation and Culture

Macrophages (*n* > 1^*^10^6^ cells per group) were isolated from the mice peritoneal as previously described with slight modifications (15). Prewarmed (37°C), 5–10 ml of 3% fetal bovine serum (FBS) were carefully injected into the peritoneal cavity. After 5 min of softly massaging the peritoneum, the fluids were collected from the cavity and centrifuged at 1,500 rpm for 10 min. The cell pellets were resuspended and cultured with 10% FBS for 48 h. Macrophage identifications were done with F4/80 (BioLegend; 123116) and CD11b (BioLegend; 101206). Subsequently, the cultured PM_Φ_ were treated with 10 μM/ml of ISO and/or 1 μg/ml LPS for 12 h, and the following treatment interventions were employed for the next 12 h. (i) 0.5 % v/v DMSO as placebo (ii) 35 μM/ml of AMLX, (iii) 10 μM/ml FSKN, and (iv) AMLX and FSKN combination, at the respective doses. These treatment interventions were explored in the absence of ISO (stress), and the dosages employed were based on their efficacy in previous studies (2, 16). Supernatants and PM_Φ_ were used for ELISA and immunofluorescence, respectively (Figure 1B).

### Immunofluorescence Staining

Fixation and permeabilization of pre-treated PM_Φ_ (*n* > 1^*^10^6^ cells per group) were done using pre-chilled methanol-acetone (ratio 1:1). The cells were blocked with 1% BSA, incubated overnight at 4°C with GRK5 antibody (ab64943; Abcam), and probed with R-PE-conjugated antibody (Proteintech; SA00008-2) at room temperature for 1 h. Next, the cells were wash and conditioned with 0.5% BSA in Hanks' balanced salt solution. Cholera toxin B (CTxB) (Thermo Fisher. C34775) was used to stain the cytoplasmic membranes for 30 min at 4°C and then counterstained with DAPI. GRK5 localization and expression ratios were ascertained using ImageJ (*n* = 12–15 cells per 4 mice).

### Statistical Analysis

Using GraphPad Prism (Prism Version 8.0.2), one-way ANOVA was used to analyze data among experimental groups and followed by Tukey's multiple comparisons test. All data were expressed as mean ± SEM. *P* < 0.05 were assigned statistical significance.

## Results

### Amlexanox and Forskolin Ameliorates Isoproterenol-Induced Left Ventricular Systolic Dysfunction

**Figure 2:**
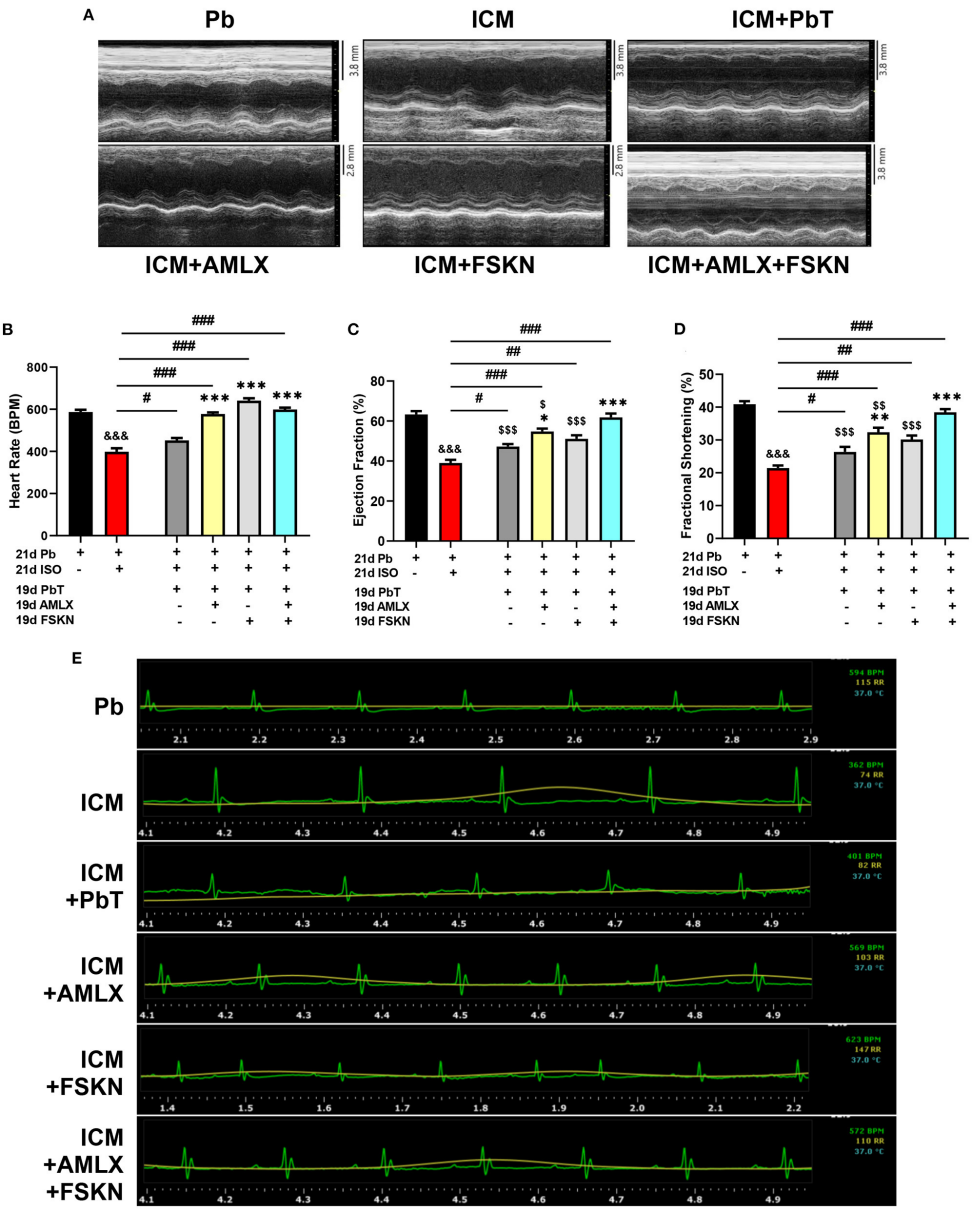
Amlexanox and Forskolin ameliorates isoproterenol-induced LVSD. **(A)** Representative echocardiogram imaging obtained in short-axis view M-mode from all the experimental groups. **(B)** Graphical presentation of heart rates assessments from electrocardiography. **(C,D)** Graphical presentation of cardiac systolic function indexes (Ejection fraction and Fractional shortening, respectively), assessed at the end of treatments (*n* = 6–8 mice per treatment group). **(E)** Representative electrocardiogram imaging from Pb, ICM, ICM+PbT, ICM+AMLX, ICM+FSKN, and ICM+AMLX+FSKN groups displaying heart rate (Green), respiration rate (yellow) and temperature (turquoise). ^&*&&*^*p* < 0.001 vs. Pb; ^#^*p* < 0.05, ^*##*^*p* < 0.01, ^*###*^*p* < 0.001; *p < 0.05, **p < 0.01, ***p < 0.001 vs. ICM+PbT; ^*$*^*p* < 0.05, ^*$$*^*p* < 0.01, ^*$$$*^*p* < 0.001 vs. ICM+AMLX+FSKN. Data are expressed as mean ± SEM. Data were analyzed using one-way ANOVA and Tukey's *post-hoc* analysis.

Results obtained from systolic function assessment using echocardiography demonstrated that the chronic administration of ISO induced LVSD as depicted in the ICM group. The LVSD observed in the ICM group is characterized by a significant decrease in heart rates (HR), EF, and FS (Figures 2A–E). Although these indexes are observed to have slightly improved in the placebo recovery treatment group (ICM+PbT), which were only given the vehicle for 19 days after the ISO injections were discontinued, cardiac functions were not fully restored as HR, EF, and FS were below their stated normal ranges (17). However, treatment of the ICM models with AMLX improved recovery from the LVSD by increase HR, EF, and FS. Contrarily, FSKN treatment results in tachycardia and arrhythmias even though EF and FS were increased in the ICM+FSKN group. Ultimately, the treatments of the ICM mice with AMLX and FSKN combination significantly enhanced the attenuation of the LVSD and hastened cardiac function recovery as HR, EF, FS, and other cardiac function indexes were all restored to their normal ranges (Figures 2A–E and Supplementary Table 1).

### Amlexanox and Forskolin Attenuates Isoproterenol-Induced Maladaptive Cardiac Hypertrophy and Promotes Fibrosis Resolution

**Figure 3:**
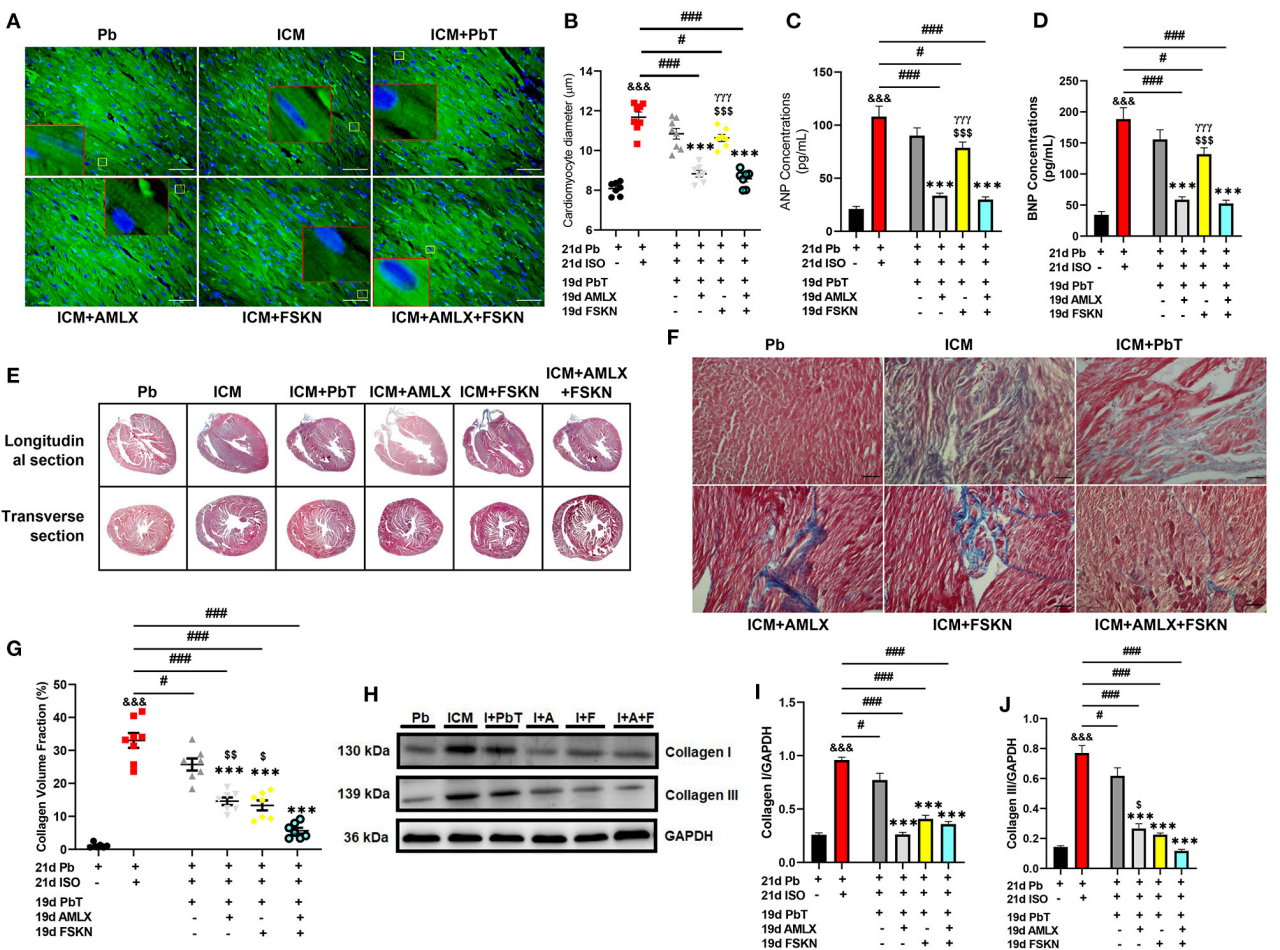
Amlexanox and Forskolin attenuates isoproterenol-induced maladaptive cardiac hypertrophy and promotes fibrosis resolution. **(A,B)** Representative wheat germ agglutinin (WGA) merged with DAPI staining and graphical presentations of measured cardiomyocyte diameter from all treatment groups (*n* = 10–12 cells per 5 field of view per 5 sections per 6–8 hearts per group). Representative cardiomyocytes are outlined in yellow boxes, and their zoomed-in (7x) inserts to show hypertrophy are outlined in red boxes. **(C,D)** Graphical plots of ELISA evaluations of cardiac hypertrophy markers (ANP and BNP, respectively), from obtained myocardial lysate. The ELISA were performed in triplicates (*n* = 6–8 mice per treatment group). **(E,F)** Representative Masson's trichrome stained longitudinal and transverse sections with detailed microscopic images showing interstitial collagen deposition. **(G)** Graphical plots collagen volume fraction from all groups (*n* = 6–8 field of view per 5–7 sections per 6–8 hearts per group). **(H–J)** Representative immunoblots and graphical presentations of Collagen I and Collagen III from Pb; placebo, ICM; isoproterenol-induced cardiomyopathy, I+PbT; ICM + placebo treatment, I+A; ICM + AMLX treatment, I+F; ICM + FSKN treatment and I+A+F; ICM + AMLX + FSKN combine treatment groups (*n* = 4 hearts per treatment group). ^&*&&*^*p* < 0.001 vs. Pb; ^#^*p* < 0.05, ^*###*^*p* < 0.001; ***p < 0.001 vs. ICM+PbT; ^γ*γγ*^*p* < 0.001 vs. ICM+AMLX; ^*$*^*p* < 0.05, ^*$$*^*p* < 0.01, ^*$$$*^*p* < 0.001 vs. ICM+AMLX+FSKN. Data are expressed as mean ± SEM. Data were analyzed using one-way ANOVA and Tukey's *post-hoc* analysis.

Cardiac morphometrics (Supplementary Table 1), histological assessment of cardiomyocyte diameter (Figures 3A,B and Supplementary Figures 1A,B), and ELISA and immunoblots of hypertrophy biomarkers (ANP and BNP) (Figures 3C,D and Supplementary Figures 1C–E) showed that chronic administration of ISO induced cardiac hypertrophy markedly. Cardiomyocyte diameters, ANP, and BNP levels were significantly increased in the ICM group. The placebo treatments (PbT) given to ICM models failed to significantly decrease the cardiomyocyte diameters after day 19; however, AMLX treatment in the AMLX+ICM group showed overt attenuation of the maladaptive cardiac hypertrophies that were observed in the ICM mice. Similarly, cardiomyocytes diameters and expression of the natriuretic peptides were observed to have decreased the FSKN treatment group, however, with lesser significance (*p* < 0.05) as compared to AMLX treatment (*p* < 0.001) and its combined treatment with FSKN (*p* < 0.001).

Also, trichrome staining of tissue sections from the ICM hearts shown typical distortions of the myocardial architecture as cardiomyocyte apoptosis and interstitial fibrosis were massively increased (Supplementary Figures 1C,F and Figures 3E–G). The placebo treatments in the ICM+PbT group showed less significance in resolving the cardiomyocyte deaths and fibrosis. However, similar to AMLX, FSKN administration to the ICM mice significantly decreased both apoptosis and collagen I and III depositions in the myocardia (Supplementary Figures 1C,F and Figures 3H–J). Intriguingly, AMLX and FSKN combination demonstrated trends of improved apoptosis attenuations and fibrosis resolutions, although there were no statistical significances compared with the single therapeutic groups.

### Amlexanox and Forskolin Enhances Attenuation of Isoproterenol-Induced Maladaptive Myocardial Inflammation

**Figure 4:**
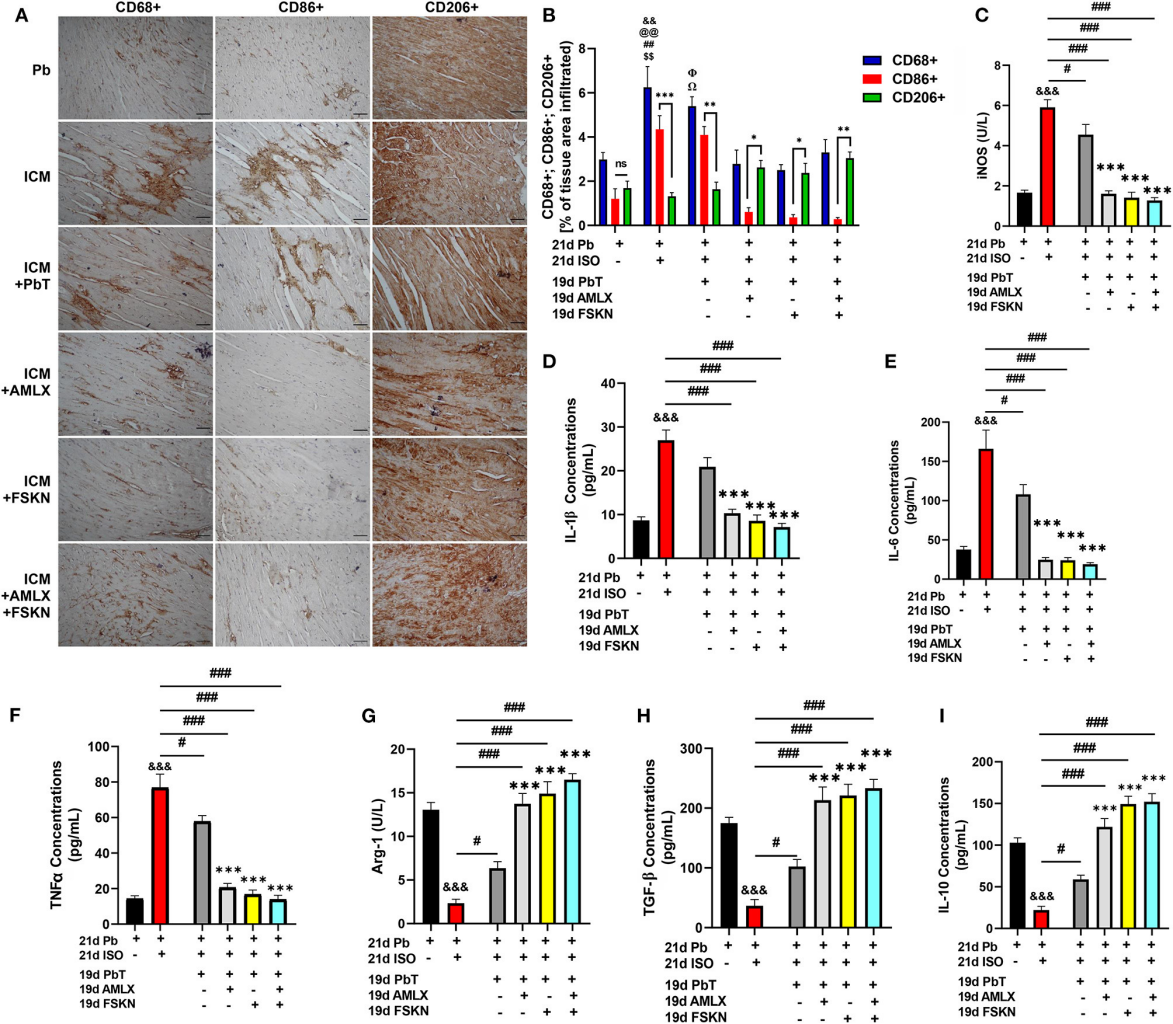
Amlexanox and Forskolin attenuates isoproterenol-induced myocardial inflammation by repressing proinflammatory mediators and responses. **(A,B)** Representative CD68+, CD86+, and CD206+ IHC staining and graphical presentations of their evaluations demonstrate the extent of inflammatory cell infiltrations into the myocardia and the ratio between pro and anti-inflammatory cells evading the myocardia among the experimental groups. ^ns^*p* ≥ 0.05, *p < 0.05, **p < 0.01, ***p < 0.001; ^&&^*p* < 0.01 vs. Pb; ^*$$*^*p* < 0.01 vs. ICM+ AMLX; ^*##*^*p* < 0.01 vs. ICM+ FSKN; Ω*p* < 0.05 vs. ICM+ AMLX; Φ*p* < 0.05 vs. ICM+FSKN; @@*p* < 0.01 vs. ICM+FSKN+AMLX **(C–F)** Graphical plots of proinflammatory mediator (iNOS) and cytokines (IL-1β, IL-6 and TNFα). **(G–I)** Graphical plots of the anti-inflammatory mediator (Arg-1) and cytokines (TGF-β and IL-10). The sera ELISA were performed in triplicates (*n* = 6–8 mice per treatment group). ^&*&&*^*p* < 0.001 vs. Pb; ^#^*p* < 0.05, ^*###*^*p* < 0.001; ***p < 0.001 vs. ICM+PbT. Data are expressed as mean ± SEM. Data were analyzed using one-way ANOVA and Tukey's *post-hoc* analysis.

IHC assessment of CD68 (total inflammatory cells), CD86 (proinflammatory cells), and CD206 (anti-inflammatory cells) from myocardial sections revealed enormous inflammatory cells infiltration into the hearts of ICM mice (Figures 4A,B). It was observed that ICM hearts had biased proinflammatory cell infiltrations while anti-inflammatory cell infiltrations were significantly reduced. After ISO discontinuation, the placebo treatment administration (in the ICM+PbT group) did not attenuate the maladaptive inflammatory responses as AMLX, FSKN and their combination treatment did. AMLX, FSKN, and their combination increase anti-inflammatory (CD206+) cells and decrease the infiltration of proinflammatory (CD86+) cells into the myocardial. ELISA of proinflammatory mediator, inducible nitric oxide synthase (iNOS) and cytokines interleukin (IL)-1β, IL-6, and tumor necrosis factor-alpha (TNFα) validated the occurrence of maladaptive inflammatory responses in the ICM model. The results also showed that AMLX and FSKN were potent at downregulating the proinflammatory responses (Figures 4C–F). Furthermore, the anti-inflammatory markers, arginase-1 (Arg-1), transforming growth factor-beta (TGF-β), and IL-10 were downregulated in ICM mice. However, AMLX and FSKN treatments upregulated these anti-inflammatory markers more significantly than was observed in the placebo treatment group (Figures 4G–I). Individually, AMLX and FSKN treatments effectively modulated inflammatory responses; however, their combination showed trends of further enhanced immunoregulatory potencies but without statistical significance.

### ALX and FSK Inhibits Proinflammatory Responses *via* G Protein-Coupled Receptor Kinase 5 Inhibition and Synergistic Upregulation of Cyclic Adenosine Monophosphate

**Figure 5:**
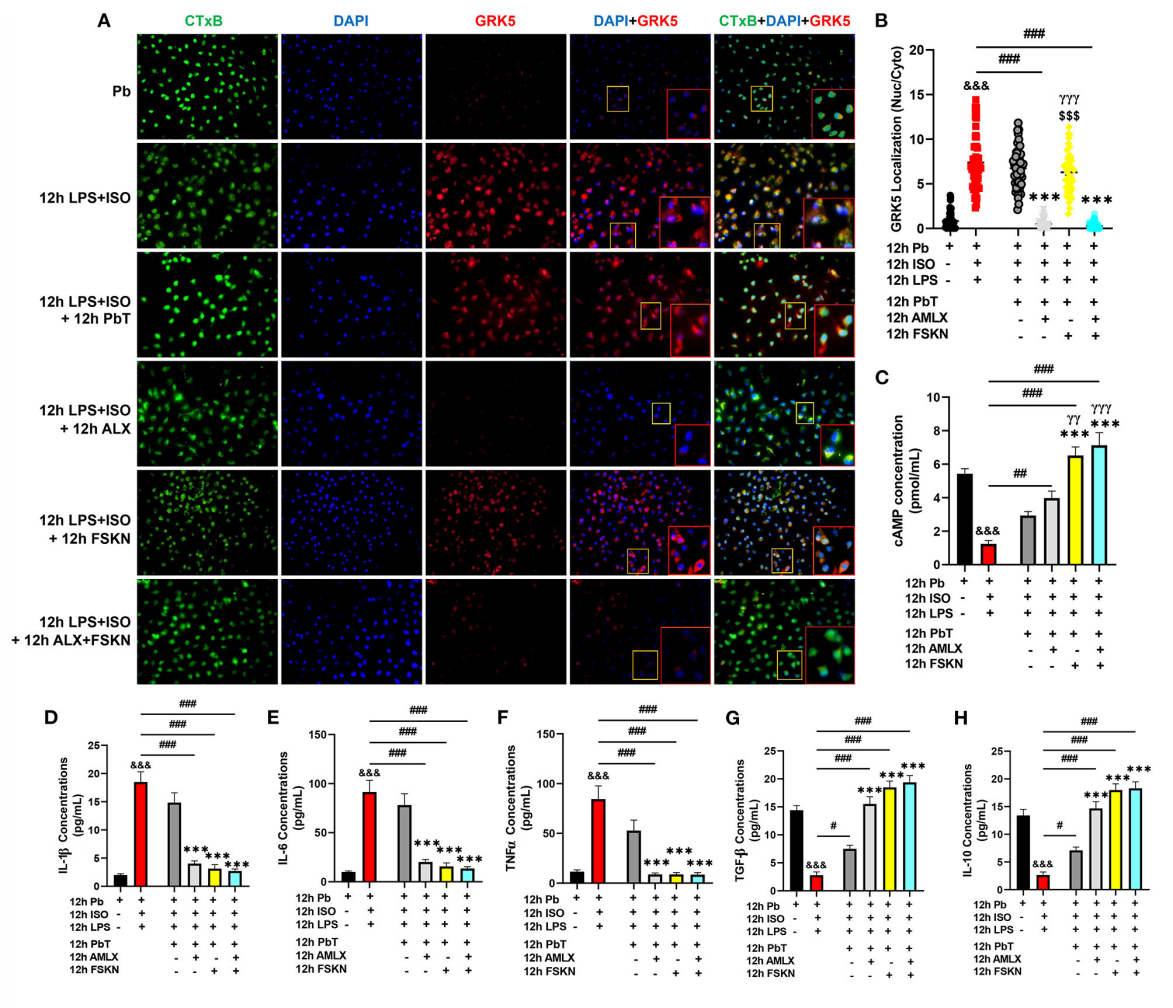
Amlexanox and Forskolin inhibits hyperactive proinflammatory responses *via* G protein-coupled receptor kinase 5 (GRK5) inhibition and synergistic upregulation of cyclic adenosine monophosphate (cAMP). **(A)** Representative immunofluorescence of GRK5 localizations, cytoplasmic membrane (CTxB), and nuclei (DAPI). **(B)** The plotted values are the GRK5 (nuclear/cytoplasm) expression ratios assessed from each PM Φ (*n* = 12–15 cells per 4 mice per group). Color channels were adjusted in the merged images to enhance the visualization of all the respective fluorescence dyes. **(C)** Graphical plots of evaluated cAMP concentrations to assess the effect of the treatment interventions cAMP bioavailability. **(D–F)** Graphical plots of ELISA evaluated proinflammatory cytokines (IL-1β, IL-6, and TNFα) concentrations. **(G,H)** Graphical presentations of ELISA evaluated anti-inflammatory cytokines (TGF-β and IL-10) concentrations. The sera ELISA were performed in triplicates (n ≥ 1*10^6^ cells per 4 mice per group). ^&*&&*^*p* < 0.001 vs Pb; ^#^*p* < 0.05, ^*##*^*p* < 0.01, ^*###*^*p* < 0.001; ***p < 0.001 vs. ICM+PbT; ^γγ^*p* < 0.01, ^γ*γγ*^*p* < 0.001 vs. ICM+AMLX; ^*$$$*^*p* < 0.001 vs. ICM+AMLX+FSKN. Data are expressed as mean ± SEM. Data were analyzed using one-way ANOVA and Tukey's *post-hoc* analysis.

Elucidations of the mechanisms employed by the treatment interventions were explored using PM_Φ_. After 12 h of ISO+LPS treatment, the PM_Φ_ were incubated with AMLX (35 μM/ml) and/or FSKN (10 μM/ml) for the next 12 h. GRK5 nucleic-cytosolic expression ratios assessed showed its upregulation and increased translocation to the nuclei after LPS+ISO treatments. Unlike the placebo (PbT) and FSKN treatment, AMLX inhibited GRK5 expression and translocation. Similarly, GRK5 activities were inhibited in the AMLX and FSKN combination treatment group (Figures 5A,B). Assessing the impact of these treatments on cAMP bioavailability demonstrated its significant upregulation in the AMLX and FSKN combination treatment group, followed by FSKN and AMLX treatments groups. cAMP concentrations were also increased in the LPS+ISO+PbT group but exhibited no statistical significance compared to the LPS+ISO group (Figure 5C). Additionally, cytokines analyses showed sustained significant upregulation of proinflammatory cytokines (IL-1β, IL-6, and TNFα) in LPS+ISO and LPS+ISO+PbT groups while the anti-inflammatory cytokines (TGF-β and IL-10) were downregulated (Figures 5D–H). Meanwhile, the contrasts of these phenomena were observed in the ISO+LPS+AMLX, ISO+LPS+FSKN, and ISO+LPS+AMLX+FSKN groups, as these treatment interventions adaptively heightened the anti-inflammatory and dampened the proinflammatory cytokines secretions. Hence, it is inferred that AMLX attained its potency via inhibiting GRK5-mediated proinflammation response activation and facilitating cAMP-mediated immunoregulation, while FSKN treatment employed only the latter. As such, the potencies attained by the AMLX and FSKN combination in the absence of stress were *via* GRK5 inhibition and the synergistic upregulation of cAMP to adaptive immunoregulatory responses.

## Discussion

Chronic stress, a resultant from the demands of our modern-day human societies, induces ICM, which ultimately results in heart failure (HF). This study aimed to explore the therapeutic potentials of AMLX in attenuating LVSD and facilitating recovery from ICM, as demonstrated in the AMI model (11). Also, we sought to, for the first time, explore if FSKN could enhance the recovery rates of the ICM model, as it stimulates the synthesis of cAMP, which is an essential modulator of cardiac and inflammatory responses (1, 2, 12, 18).

The results obtained from cardiac function assessments using ECG and ECHO revealed that HR, EF, and FS were significantly reduced in the ICM groups, which are indicative of the occurrence of LVSD. Comparing the obtained values for these clinically relevant cardiac function indexes with other similar studies (19), it could be remarked that the hearts of mice from the ICM group were progressing into HF. The administration of 5%v/v DMSO as a placebo treatment for 19 days after discontinuing ISO injection did not restore HR, EF, and FS to their normal ranges. However, AMLX treatment improved HR and systolic functions, which are consistent with Mo et al.'s findings (11). Nonetheless, our previous study found that when AMLX treatments were given simultaneously with ISO injections, AMLX's efficacy in attenuating LVSD was dampened (2). Hence, it is speculated that for AMLX treatment to be effective at attenuating LVSD in ICM, the sources of stressors must be obliterated. Also, FSKN treatments resulted in tachycardia and arrhythmias. In conformity with these findings, Christ et al. and Huang et al. had early demonstrated the arrhythmic side effects of FSKN treatment (20, 21). Intriguing, AMLX, and FSKN combination demonstrated the most potency in restoring systolic functions in the ICM mice.

Furthermore, assessments of cardiac morphometric data, cardiomyocyte diameter from histological analysis, and expressions of hypertrophy biomarkers (ANP and BNP) provided additional evidence that ICM is characterized by a pathologically hypertrophied heart (8, 22). Again, treatment of the ICM mice with placebo demonstrated an insignificant decrease in cardiomyocytes' diameters. In contrast, AMLX treatment showed overt attenuation of the hypertrophy, which were validated as the natriuretic peptides ANP and BNP expressions well also downregulated, as previously reported (2, 23, 24). Consistent with the findings of Gesmundo and Miragoli (25), FSKN treatment *via* AC/cAMP/PKA exerted slight anti-hypertrophic effects, but these were less significant as compared to AMLX treatment. Hence, the AMLX and FSKN combine treatment attenuated maladaptive cardiac hypertrophy in ICM mice synergistically.

Clinically, the LVSD occurring in patients with ICM is attributed to increased interstitial fibrosis, which disrupts the typical myocardial architecture and causes stiffness of the heart (7, 26). The increased fibrosis ultimately impedes the heart from rapidly replenishing a sufficient amount of blood for the subsequent ejection (27). In conformity with the characterization of the myocardial remodeling occurring in ICM patients, histological assessments of hearts from the ICM models showed the occurrence of massive interstitial fibrosis. Therefore, the synergy of maladaptive ventricular hypertrophy and increased fibrosis in the ICM heart might constitute underlying factors contributing to the LVSD. Individually, AMLX and FSKN treatments promoted myocardial fibrosis resolution effectively compared to the placebo (in ICM+ PbT) (Figures 3E–G). Zhou et al. had previously shown the anti-fibrotic potencies of AMLX treatment (28). Similarly, consistent with our findings, FSKN treatments were shown by El-Agroudy et al. and Roberts et al. to exert anti-fibrotic effects by inhibiting fibroblast activation, proliferation, and differentiation (29, 30). As such, by coupling their anti-fibrotic effects, AMLX and FSK combination further enhanced myocardial fibrosis resolution significantly, as evidenced by the more decreases in collagen expression and deposition (Figures 3E–J).

Recent therapeutic interventions aimed at attenuating pathological remodeling of the heart are targeted at the adaptive modulation of myocardial inflammatory responses, besides preserving and sustaining cardiomyocyte function (11). This is due to the fact that maladaptive myocardial inflammatory responses have been implicated in expediting the adverse remodeling of the heart in most CVDs (1, 11, 31, 32). Therefore, the myocardia of ICM mice were histologically assessed to ascertain the extent of inflammatory cell infiltrations. As demonstrated in the aforementioned studies, enormous amounts of inflammatory cells (CD68 positive) were found in the ICM hearts, of which the majority were CD86 positive cells and accompanied by fewer CD206 positive cells. Individually, AMLX and FSKN treatments administered to the ICM models exert adaptive immunoregulatory effects in the myocardia. Both significantly downregulated CD86 positive cells and comparatively increased CD206 positive cells infiltration to facilitate timely resolution of the myocardial inflammation. In addition, it was observed that the AMLX and FSKN treatments decreased iNOS, IL-1β, IL-6, and TNFα while upregulating Arg-1, TGF-β, and IL-10. Likewise, their combination treatment synergistically exerted anti-inflammatory effects to timely resolve the observed myocardial inflammation in the ICM models. In conformity with these findings, previous studies have extensively demonstrated the anti-inflammatory effects of AMLX and FSKN (11, 18, 33). Again, it is worth mentioning that, in our previous study, where AMLX treatments were administered during ISO-induced stress, it failed to facilitate anti-inflammatory responses effectively (2). As such, the elimination of stressors is crucial as they affect the anti-inflammatory potency of AMLX.

Previously, we had reported that rather than the individual treatments of AMLX and FSKN, their combination was the most potent treatment intervention for preventing ICM during chronic stress (2). However, findings from this explorational study which sought to find the therapeutic potentials of AMLX and FSKN in promoting recovery from ICM, showed the following. (1) Comparatively, AMLX treatment improved cardiac functions in the ICM recovery model but failed to sustain these functions during stress in ICM preventive models. (2) AMLX treatment had failed to inhibit the upregulation of proinflammatory responses in ICM preventive models during chronic catecholamine stress but comparably exerted adaptive immunoregulation in the myocardial of ICM recovery models in the absence of stress. (3) Lastly, although AMLX and FSKN combination treatment showed increased adaptive immunoregulation trends, there were no statistical significance among it and the individual treatments with AMLX and FSKN.

Hence, further experiments were performed to elucidate the possible underlying mechanisms for the observed outcomes. By mimicking cardiac damage-associated molecular patterns with LPS during stress, hyperactive proinflammatory responses were elicited from PM_Φ_. The GRK5 nucleic–cytosolic expression ratios assessed in PM_Φ_ after followed-up treatments with AMLX and FSKN and their combination showed similar results as previous (2). Only AMLX in both single and combination treatment repressed GRK5 expression and translocation, while FSKN failed to do these similarly to the placebo. Additionally, evaluated cAMP concentration from the culture supernatants after the treatment intervention showed significant upregulations in AMLX and FSKN and their combination treatment groups, but not in the placebo (ISO+LPS+PbT) group. While FSKN is well-demonstrated to facilitate cAMP synthesis by directly activating adenylyl cyclase activity (20, 34), finding cAMP bioavailability being facilitated by AMLX was intriguing as this phenomenon was observed previously under catecholamine stress condition (2). However, consistent with the finding, Han et al. had previously shown that besides GRK5, AMLX non-selectively inhibits phosphodiesterase (PDE), which degrades cAMP (35). As such, by impeding the degradation of cAMP by PDE, AMLX sustained cAMP bioavailability in the absence of stress, as demonstrated here. Followed-up cytokines analyses revealed the effective attenuation of proinflammatory responses (IL-1β, IL-6, and TNFα) and the upregulation of TGF-β and IL-10 by AMLX and FSKN and their combination treatment. Therefore, inferences made from the findings in Figure 5 confirm that ALX and FSK treatment enhanced the resolution of maladaptive myocardial inflammation by inhibiting GRK5-mediated inflammation and synergistic enhancement of cAMP bioavailability which exerted adaptive immunoregulation and promoted recovery from ICM.

Taken together, the findings from this study demonstrate that treating ICM models with AMLX and FSKN combination enhances the recovery outcomes by attenuating LVSD and timely resolving both myocardial inflammation and fibrosis. However, the source of stressors must be eliminated during the recovery treatment as it undermines the efficacy of AMLX. Also, due to the clinical significance of this study, it is recommended that the toxicity of AMLX and FSKN combination is determined, and its effect on diastolic function and lung congestion be evaluated to ascertain their therapeutic and translational potentials fully.

## Data Availability Statement

The original contributions presented in the study are included in the article/Supplementary Material, further inquiries can be directed to the corresponding author/s.

## Ethics Statement

The animal study was reviewed and approved by Experimental Animal Centre of Xuzhou Medical University and the Animal Ethics Committee of the Medical University (permit number: xz11-12541).

## Author Contributions

GKA conceived the experiment ideas. With HS's supervision, GKA designed the experiments. HJH and AOA provided experimental animals. RR, JA-A, and WS assisted in making animal models. KL and Q-MD performed cardiac function assessments. GKA, JA-A, RM, and MLNN performed furthers experiments. GKA, WS, and Q-MD analyzed and interpreted data. GKA wrote the manuscript based on contributions from all authors. GKA, HJH, AOA, RR, JA-A, WS, RM, and MLNN proofread and all authors approved the manuscript.

## Funding

This research was supported by National Natural Science Foundation of China (Grant Nos. 81461138036 and 81370329), Postgraduate Research & Practice Innovation Program of Jiangsu Province, China (KYCX17-1712), and Priority Academic Program Development of Jiangsu Higher Education Institutions (PAPD).

## Conflict of Interest

The authors declare that the research was conducted in the absence of any commercial or financial relationships that could be construed as a potential conflict of interest.

## Publisher's Note

All claims expressed in this article are solely those of the authors and do not necessarily represent those of their affiliated organizations, or those of the publisher, the editors and the reviewers. Any product that may be evaluated in this article, or claim that may be made by its manufacturer, is not guaranteed or endorsed by the publisher.

## Abbreviations

AC: Adenylyl cyclase
AMLX: Amlexanox
cAMP: cyclic adenosine monophosphate
FSKN: Forskolin
GRK: G protein-coupled receptor kinases
ISO: Isoproterenol
LPS: Lipopolysaccharide
LVSD: Left ventricular systolic dysfunction
PM_Φ_: Peritoneal macrophage
PbT: Placebo treatment.
