# Supplementary material for: Isoproterenol-Induced Cardiomyopathy Recovery Intervention: Amlexanox and Forskolin Enhances the Resolution of Catecholamine Stress-Induced Maladaptive Myocardial Remodeling
Source: Front Cardiovasc Med. 2021 Nov 25;8:719805. doi: 10.3389/fcvm.2021.719805 (PMC8655225; doi:10.3389/fcvm.2021.719805)
Supplement: Supplementary file 1 [file Data_Sheet_1.docx]

**Figure S1: (A and B)** Representative H&E staining images and graphical presentations of measured cardiomyocyte diameter from all treatment groups (n=10-12cells per 5 field of view per 5 sections per 6-8 hearts per group). **(C and E)** Representative immunoblots and graphical presentations of ANP, BNP and Cleaved Caspase-3 from Pb; placebo, ICM; isoproterenol-induced cardiomyopathy, I+PbT; ICM + placebo treatment, I+A; ICM + AMLX treatment, I+F; ICM + FSKN treatment and I+A+F; ICM + AMLX + FSKN combine treatment groups (n=4 hearts per treatment group). &&&p<0.001 vs Pb; #p<0.05, ##p<0.01, ###p<0.001; ***p<0.001 vs ICM+PbT; **γγγ** p<0.001 vs ICM+AMLX; $$$p<0.001 vs ICM+AMLX+FSKN. Data are expressed as mean ± SEM. Data were analyzed using one-way ANOVA and Tukey’s post hoc analysis.

**A**

**B**

**Fig. S1**

**Pb ICM ICM+PbT**


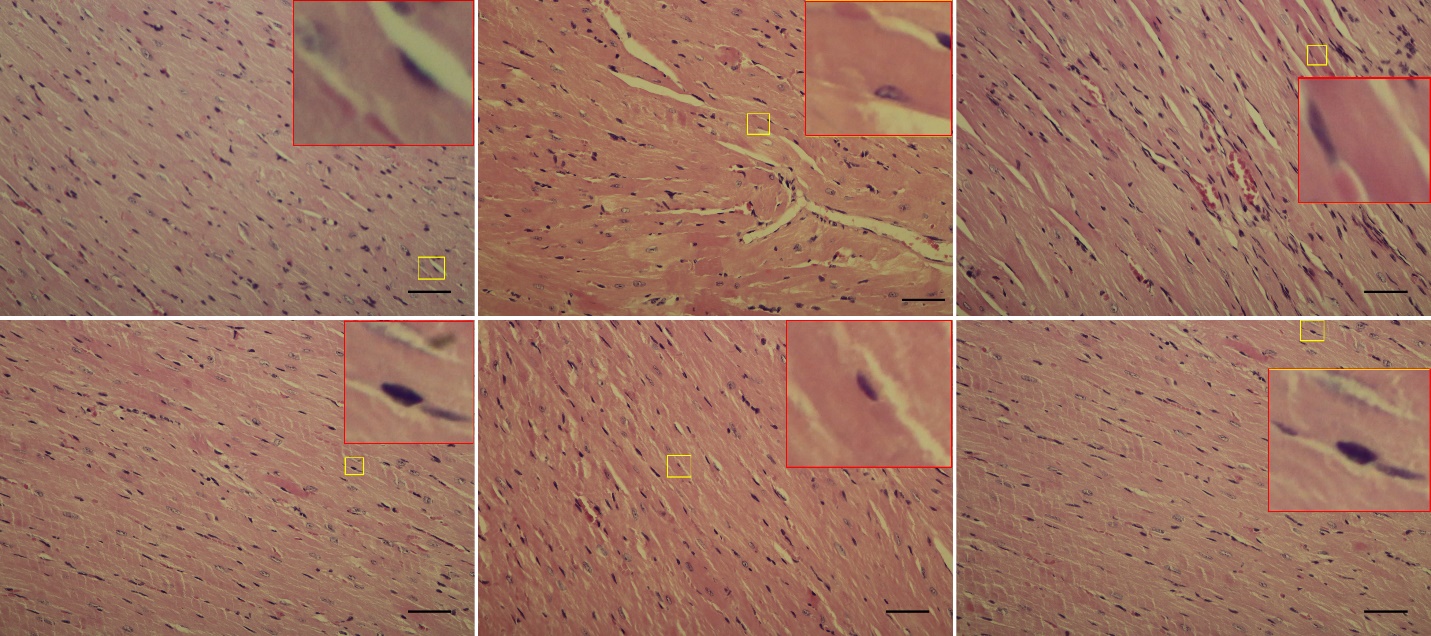


**ICM+AMLX ICM+FSKN ICM+AMLX+FSKN**

**C**

**E**

**F**

**D**

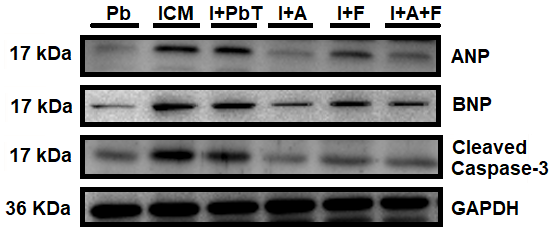


**Table S1:** **Quantitative data of morphometry, echocardiography and electrocardiography assessments**

| Parameter | Pb | ICM | ICM+Pb | ICM+ALX | ICM+FSK | ICM+ALX+FSK |
| --- | --- | --- | --- | --- | --- | --- |
| Morphometry | | | | | | |
| *n* (hearts) | 6 | 8 | 8 | 8 | 8 | 8 |
| HW/BW (mg/g) | 4.35 ± 0.15 **&&&** | 5.37 ± 0.22 | 5.25 ± 0.57 | 4.38 ± 0.15*****###** | 5.08 ± 0.2 ***##** | 4.39 ± 0.10*****###** |
| LVM (mg) | 83.39 ± 0.22 **&&&** | 130.13 ± 0.47 | 124.55 ± 0.61 | 84.83 ± 0.32*****###** | 122.52 ± 0.47 ***#** | 85.33 ± 0.41*****###** |
| Echocardiography | | | | | | |
| *n* (hearts) | 6 | 8 | 8 | 8 | 8 | 8 |
| IVS; d (mm) | 0.728 ± 0.06 **&&&** | 0.858 ± 0.17 | 0. 843 ± 0.21 | 0.718 ± 0.07*****###** | 0.803 ± 0.13 ****###** | 0.722 ± 0.17*****###** |
| IVS; s(mm) | 1.100 ± 0.08 **&&&** | 1.318 ± 0.15 | 1.310 ± 0.13 | 1.122 ± 0.14*****###** | 1.275 ± 0.08****###** | 1.121 ± 0.11*****###** |
| LVID; d (mm) | 3.735 ± 0.10 **&&&** | 4.853 ± 0.45 | 4.572 ± 0.40**^** | 4.148 ± 0.58*****###** | 4.330 ± 0.63 ****###** | 3.977 ± 0.23*****###** |
| LVID; s (mm) | 2.390 ± 0.28 **&&&** | 3.843 ± 0.33 | 3.600 ± 0.43**^** | 2.933 ± 0.61*****###** | 3.229 ± 0.42****###** | 2.502 ± 0.27*****###** |
| LVPW; d (mm) | 0.740 ± 0.08 **&&&** | 1.015 ± 0.13 | 1.08 ± 0.10 | 0.763 ± 0.20*****###** | 0.846 ± 0.23 ***##** | 0.769 ± 0.21*****###** |
| LVPW; s (mm) | 1.025 ± 0.18 **&&&** | 1.456 ± 0.21 | 1.444 ± 0.32 | 1.097 ± 0.12*** **###** | 1.163 ± 0.19 ***##** | 1.061 ± 0.03*****###** |
| Electrocardiography | | | | | | |
| *n* (hearts) | 6 | 8 | 8 | 8 | 8 | 8 |
| QRS Interval (s) | 0.00958 ± 0.00075**&&&** | 0.0130 ± 0.00048 | 0.0123±0.00053**^** | 0.0101±0.0001****###** | 0.0916±0.00018*****###** | 0.00938±0.00010*****###** |
| QT Interval (s) | 0.020 ± 0.002 **&&&** | 0.035 ± 0.010 | 0.030 ± 0.007**^** | 0.027 ± 0.0009****###** | 0.018 ± 0.002 *****###** | 0.019± 0.001*****###** |
| QTc (s) | 0.053 ± 0.003 **&&&** | 0.080 ± 0.009 | 0.076 ± 0.008**^** | 0.064 ± 0.007****###** | 0.053 ± 0.004 *****###** | 0.054 ± 0.006*****###** |
| T Amplitude (mV) | 0.478 ± 0.040 **&&&** | 0.193 ± 0.053 | 0.288 ± 0.074**^** | 0.363 ± 0.056*****###** | 0.482 ± 0.031 *****###** | 0.462 ± 0.021*****###** |
| ST Height (mV) | 0.39 ± 0.070 **&&&** | 0.17 ± 0.064 | 0.23 ± 0.010**^** | 0.27 ± 0.048*****###** | 0.21 ± 0.014**##** | 0.35 ± 0.051*****###** |
| JT Interval (s) | 0.00757 ± 0.00053 **&&&** | 0.02434 ± 0.00018 | 0.02001±0.0020**^** | 0.01306±0.0017*****###** | 0.00684±0.0021*****###** | 0.00712±0.00049*****###** |
| P Amplitude (mV) | 0.08890 ± 0.012 **&&&** | -0.1027 ± 0.0051 | 0.04011±0.030**^** | 0.06453±0.060*****###** | 0.0543±0.0034****###** | 0.08302±0.0017*****###** |
| PR Interval (s) | 0.03844 ± 0.00013 **&&&** | 0.04373 ± 0.00060 | 0.04073±0.00090**^** | 0.03988±0.00011**###** | 0.03740±0.0012*****###** | 0.03789±0.0036*****###** |

**Table S1:** **HW**, heart weight; **BW**, body weight; **LVM**, left ventricle mass; **IVSd**, Interventricular septal thickness diastolic; **IVSs**, Interventricular septal thickness systolic; **LVIDd**, left ventricular internal diameter diastolic; **LVIDs**, left ventricular internal diameter systolic; **LVPWd**, left ventricular posterior wall thickness diastolic; **LVPWs**, left ventricular posterior wall thickness systolic; **QTc**, Corrected QT Interval. Data are expressed as mean ± SD. P values < 0.05 were deemed significant. **&&&**p<0.001 Pb vs ICM; **^**p<0.05 ICM+Pb vs ICM; *p<0.05, **p<0.01 PCH, ***p<0.001 vs ICM+PbT; #p<0.05, ##p<0.01 PCH, ###p<0.001 vs ICM.
